# Fabrication of Co-Assembly from Berberine and Tannic Acid for Multidrug-Resistant Bacteria Infection Treatment

**DOI:** 10.3390/pharmaceutics15071782

**Published:** 2023-06-21

**Authors:** Tingting Zheng, Huan Chen, Chenyang Wu, Jinrui Wang, Mengyao Cui, Hanyi Ye, Yifan Feng, Ying Li, Zhengqi Dong

**Affiliations:** 1 Drug Delivery Research Center, Institute of Medicinal Plant Development, Chinese Academy of Medical Sciences, Peking Union Medical College, Beijing 100193, China; s2020009014@student.pumc.edu.cn (T.Z.); 15030413919@163.com (H.C.); wuchenyang54@163.com (C.W.); 15776622452@139.com (J.W.); cuimengyao5521@163.com (M.C.); yehy6620@163.com (H.Y.); fyf5501@163.com (Y.F.); 2 Key Laboratory of Bioactive Substances and Resources Utilization of Chinese Herbal Medicine, Ministry of Education, Chinese Academy of Medical Sciences, Peking Union Medical College, Beijing 100094, China; 3 Key Laboratory of New Drug Discovery Based on Classic Chinese Medicine Prescription, Beijing 100700, China; 4 Beijing Key Laboratory of Innovative Drug Discovery of Traditional Chinese Medicine (Natural Medicine) and Translational Medicine, Beijing 100700, China

**Keywords:** co-assembly, berberine, tannic acid, multidrug-resistant bacterial infections

## Abstract

Long-term antibiotic use induces drug resistance in bacteria. This has given rise to the challenge of refractory infections, which have become a global health threat. Berberine (BBR) and tannic acid (TA) from plants exhibit promising antibacterial activities and may overcome antibiotic resistance. However, poor solubility and/or low penetration capability have limited their application. Carrier-free co-assembled nanocomposites composed entirely of BBR and TA exhibit improved or new properties and produce improved efficacy. Herein, we demonstrated that an ordered nanostructure could be spontaneously co-assembled by the solvent evaporation method using the two natural products. These co-assembled berberine–tannic acid nanoparticles (BBR-TA NPs) exhibited the best antibacterial effect compared with the corresponding physical mixture, pristine BBR, and some first-line antibiotics (benzylpenicillin potassium-BP and ciprofloxacin-Cip) against *Staphylococcus aureus* (*S. aureus*) and *multidrug-resistant Staphylococcus aureus* (*MRSA*). Even if the concentration of BBR-TA NPs was as low as 15.63 μg/mL, the antibacterial rate against *S. aureus* and *MRSA* was more than 80%. In addition to the synergistic effect of the two compounds, the antibacterial mechanism underlying the nanostructures was that they strongly adhered to the surface of the bacterial cell wall, thereby inducing cell membrane damage and intracellular ATP leakage. Furthermore, the in vivo wound healing effect of BBR-TA NPs was verified using an *MRSA* wound infection mouse model. The BBR-TA NPs achieved the best efficacy compared with BP and Cip. Moreover, cytotoxic and histopathological evaluations of mice revealed that the nanodrug had good biological safety. This facile and green co-assembly strategy for preparing nanoparticles provides a feasible reference for the clinical treatment of bacterial infection.

## 1. Introduction

Infectious diseases caused by bacteria are a serious threat to public health and safety [1]. Since the discovery of penicillin as a drug that kills staphylococci in 1929, the field has gradually entered the era of the control and treatment of bacterial infectious diseases [2]. However, the increased unnecessary use of antibiotics has accelerated the evolution of pathogenic bacteria. Therefore, numerous drug-resistant bacteria and multidrug-resistant (MDR) bacteria have continued to emerge in recent years. The antibacterial mechanism has evolved various immune mechanisms, including both congenital and adaptive [3]. Antibiotic resistance has given rise to the challenge of refractory infections [4,5], and the emergence of MDR bacteria has become a global health threat [6]. In the United States, the resistance rate of Enterobacteriaceae can reach up to 88%. In India, the resistance rate of Escherichia coli can reach up to 92% [7]. China is currently the largest user of antibiotics in the world, accounting for more than half of the world’s prescriptions. It is, thus, also one of the countries most affected by antibiotic resistance [8]. With the increase in the incidence of this serious phenomenon of bacterial drug resistance, the discovery of new antimicrobial agents is imminent [9]. Therefore, new drugs with low toxicity and side effects against drug-resistant bacteria need to be urgently developed.

The field of nanotechnology-based drugs that combat multidrug-resistant microorganisms has made significant progress [10,11]. Different types of nano-antimicrobial agents have been investigated to improve the efficiency of antibacterial treatment [12,13]. Collectively, the antibacterial efficacy of nanodrugs depends on the physical and chemical properties of nanoparticles (NPs). Small NPs easily penetrate bacterial cells. NPs with a positive zeta potential can react with negatively charged bacterial surfaces via electrostatic interactions, leading to membrane disruption. NPs can further disrupt DNA to inhibit bacterial replication and cell division [14]. NPs can also produce a larger contact area with bacteria, thereby enhancing the antibacterial activity [15,16]. NPs have great potential for preventing and treating microbial infections; however, their safety and cytotoxicity have become major obstacles to clinical transformation. Polymer chemistry-based NPs usually have a complicated manufacturing process and a low drug-loading capacity. They also induce toxicity and inflammation during degradation and metabolism in humans [17,18,19]. Thus, a supramolecular assembly composed entirely of pharmacologically active molecules needs no carriers, and the reaction conditions are simple and economical. This allows a polymer-free and facile construction of biocompatible NPs for clinical applications [20].

Nanocomposites co-assembled with different molecules exhibit improved or new properties that can be exploited for multifunctional applications. Thus, the co-administration of drug combinations has been shown to produce improved efficacy over the administration of a free drug. Studies have recently shown that various alkaloids [21,22,23] and phenolic compounds [24,25,26,27] display good bacteriostatic activity. Plant-derived polyphenols have been greatly welcomed as antimicrobial agents. Tannic acid (TA) has a wide range of pharmacological activities, such as antibacterial, anti-inflammatory, neuroprotective, anti-tumor, cardioprotective, and anti-pathogenic effects [28]. TA interferes with cell metabolism, causing cell destruction; inhibits bacterial attachment to surfaces, resulting in bacterial cell death; and inhibits sugar and amino acid uptake, limiting bacterial growth [29,30]. However, the use of TA in this field of antibacterial research has been limited, one of the main reasons being the limited efficiency of TA to cross the bacterial membrane [31]. Berberine (BBR), a natural isoquinoline alkaloid, has many biological functions such as anti-inflammatory [32], anti-infection [33], and anti-arrhythmia [34] effects; diabetes treatment [35]; and the regulation of blood lipid and blood glucose. In particular, BBR has been developed as a first-line drug for the treatment of bacterial diarrhea [36,37]. BBR can inhibit DNA duplication, RNA transcription, protein biosynthesis, and enzyme activities and destroy the bacterial cell surface structure [38]. However, BBR has poor solubility and a low tendency for drug resistance [39], which restricts its extensive use in battling bacterial infections. Both BBR and TA have complex aromatic ring structures. We speculate that the co-assembly strategy can cause BBR and TA to form a new configuration. Thus, this strategy can improve the solubility of these compounds and their adhesion to bacteria, further resulting in synergistic interactions for improving MDR bactericidal efficacy based on their different antibacterial mechanisms.

**Scheme 1:**
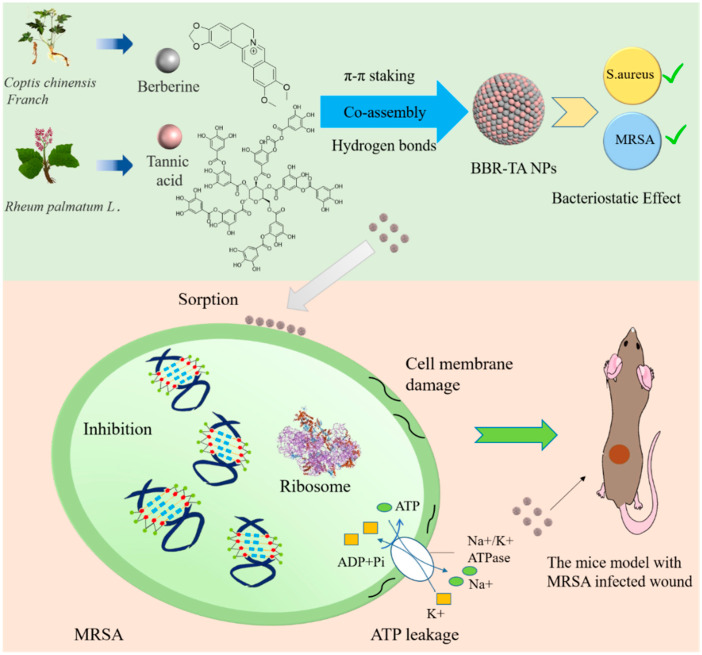
Schematic illustration of co-assembly and antibacterial mechanism of BBR-TA NPs.

Based on this hypothesis, we determined whether the two natural products can co-assemble to form an ordered nanostructure and exert a better antibacterial effect. The preparation of berberine–tannic acid nanoparticles (BBR-TA NPs) was carried out by the solvent evaporation method. Although there are many methods for preparing nanoparticles, such as the spontaneous emulsion solvent diffusion method, salting-out emulsion diffusion method, nanoprecipitation method, etc., the solvent evaporation method is the simplest method, and nanoparticles with a small size and good dispersion are obtained. The results revealed that the nanostructures could be co-assembled at a 1:1 molar ratio. These nanostructures had the best antibacterial effect compared with other physical mixtures and crude compounds used against Gram-positive bacteria (e.g., *Staphylococcus aureus*-*S. aureus* and *Multidrug resistant Staphylococcus aureus*-*MRSA*). In addition to the synergistic effect exerted by the two compounds, the antibacterial action mechanism of the nanostructures was that they more easily adhered to the surface of the bacterial cell wall. Furthermore, the in vivo bacteria-infected wound healing effect of BBR-TA NPs was verified using an *MRSA*-infected mouse model, and they displayed better efficacy than BBR, TA, and first-line antibiotics (benzylpenicillin potassium-BP and ciprofloxacin-Cip). BBR-TA NPs thus benefited from the superiority of nanomedicine and combination therapy. This design acts as a feasible reference for developing non-antibiotic drugs based on natural products to combat antibiotic-resistant bacterial infections. The schematic illustration of the co-assembly and the antibacterial mechanism of the BBR-TA NPs is presented in Scheme 1.

## 2. Materials and Methods

### 2.1. Materials

Berberine was purchased from Shanghai Ronghe Pharmaceutical Technology Development Co., Ltd. (Shanghai, China), and tannic acid was purchased from Sigma-Aldrich Trading Co., Ltd. (Shanghai, China). Benzylpenicillin potassium and ciprofloxacin were purchased in Shanghai Macklin Biochemical CO., Ltd. (Shanghai, China). *S. aureus* was purchased from China’s Center of Industrial Culture collection. *MRSA* was provided by the Laboratory Department of Dongzhimen Hospital, Beijing University of Traditional Chinese Medicine (sample number: 19PXTH0119). The LIVE/DEAD bacteria staining kit was purchased from Thermo Fisher Scientific (Waltham, MA, USA). The ATP detection kit was purchased from Beyotime Institute of Biotechnology (Shanghai, China).

### 2.2. Preparation of BBR-TA NPs

An appropriate amount of BBR was weighed to prepare different concentrations (0.5 mg/mL, 1 mg/mL, and 2 mg/mL) of berberine ethanol solution. An appropriate amount of tannic acid was weighed to prepare different concentrations (2 mg/mL, 4 mg/mL, and 8 mg/mL) of tannic acid aqueous solution. After vortex oscillation for 1 min, the two solutions were fully dissolved by ultrasound for 10 min. Berberine ethanol solution was added drop by drop into tannic acid aqueous solution (volume ratio 1:1) with a syringe and stirred for 2 h at room temperature to prepare nanoparticles with different ratios (molar ratio: 1:1, 1:2, 2:1, 1:4, and 4:1). After freeze-drying (−80, 48 h), the nanoparticles were redissolved with water. Then, dialysis was performed with ultra-pure water for 6–8 h to remove free drugs, and the nanoparticles were freeze-dried again to obtain BBR-TA NPs. The freeze-dried nanoparticles were stored at 4 °C before use.

### 2.3. Characterization of BBR-TA NPs

The size and zeta potential of BBR-TA NPs with different ratios were characterized using the Zetasizer Nano ZS (Malvern Instrument, Malvern, Worcestershire, UK). The morphological images of co-assembled nanostructures were obtained using transmission electron microscopy (TEM, G2 F30, FEI, Hillsboro, OR, USA). The particle size was determined using the dynamic light scattering method at room temperature. ^1^H nuclear magnetic resonance (600 MHz AVANCE III, Bruker, Karlsruhe, Germany) was used to visualize the formation, conformation, and co-assembly mechanism of the complex unit. The phase change before and after NP formation was determined using X-ray diffraction (XRD; D8 ADVANCE, Bruker, Karlsruhe, Germany).

### 2.4. Molecular Dynamics Simulations

The initial configurations of the systems were constructed using the Packmol software. The simulations were performed using GROMACS package (version 2019.3) with the all-atom OPLS (optimized performance for liquid systems) force field. For the system, the steep descent method was used to minimize the energy of the system. Then, molecular dynamics simulations under NPT ensemble at 298 K and 1 atm were performed for 20 ns for the system to collect trajectories for the subsequent data analysis. LINCS algorithm was applied to constrain the bond lengths of other components. Periodic boundary conditions were applied in all three directions. The temperature was maintained using the V-rescale thermostat algorithm. The cut-off distance for the Lennard-Jones and electrostatic interactions was 1.2 nm. Particle mesh Ewald method was used to calculate the long-range electrostatic interactions. Configurations were visualized using Visual Molecular Dynamics software (VMD version 1.9.2, University of Illinois, Champaign-Urbana, IL, USA).

### 2.5. Bacteria and Culture Conditions

*S. aureus* and *MRSA* in 50% glycerol medium were transferred to the liquid medium of nutritious gravy and broth and cultured at 37 °C for 16 h. Bacterial concentration during interaction with drugs was determined using the plate counting method.

### 2.6. Determination of Inhibition Ratio

Fluorescence staining method: *S. aureus* and *MRSA* were dispersed in the liquid medium of nutritious gravy and broth. The bacterial cells were rotated and cultured overnight at 37 °C and 200 rpm to attain the logarithmic phase. BBR, TA, BBR/TA MIX, BBR-TA NPs, BP, and Cip with a concentration gradient were prepared in water. Then, 500 μL each of the drug solution and the bacterial suspension was added to a centrifuge tube and cultured at 37 °C and 200 rpm for 3 h. The bacteria were stained using the LIVE/DEAD BacLight dye (LIVE/DEADTM BacLightTM Bacterial Viability kit for microscopy and quantitative assays, L7012) and incubated for 15 min. Fluorescence was measured at excitation/emission wavelengths of 485 nm/542 nm, respectively, using the multifunctional microplate detector. The inhibition ratio was calculated as follows:

Inhibition ratio (%) = (OD_control_ − OD_sample_) × 100/OD_control_% (1)

### 2.7. Bacterial Cell Membrane Integrity

The percentage of bacterial membrane integrity was determined using the LIVE/DEAD bacteria detection kit. BBR, TA, BBR/TA MIX, and BBR-TA NPs were added to the logarithmic phase bacterial suspension and incubated at 37 °C for 3 h. After incubation, the bacterial cells were collected, washed, and re-suspended with the same amount of 0.9% sodium chloride. The treated bacterial cells (100 μL) were mixed with 100 μL of working dye solution in black and opaque 96-well microtitration plates. After incubating the cells for 15 min at room temperature, the membrane integrity was observed under a fluorescence microscope (eclipse ts2R, Nikon, Tokyo, Japan). A drop of 20 μL of suspension was placed on the slide’s surface and covered with the cover slide, and the fluorescence image was immediately obtained.

### 2.8. Visual Assay of the Scavenging Effect of Biofilm

*S. aureus* and *MRSA* in the logarithmic growth phase were inoculated in a 48-well plate with cell climbing tablets at the bottom and incubated for 24 h to form a complete biofilm. Then, the culture medium was completely removed, and BBR, TA, BBR/TA MIX, BBR-TA NPs, BP, and Cip were added to the 48-well plate. After incubating at 37 °C for 24 h, they were washed with PBS three times. Then, the biofilm was fixed with 2.5% glutaraldehyde for 4 h and dehydrated with increasing ethanol concentrations (30%, 50%, 70%, 80%, 90%, 95%, and 100%) for 10 min. After lyophilization and dehydration, the biofilm was observed using scanning electron microscopy (SEM).

### 2.9. Determination of Intracellular ATP in Bacteria

The ATP concentration was determined using the ATP detection kit (Biyuntian (Shanghai, China), S0027). BBR, TA, BBT/TA MIX, and BBR-TA NPs were added to the logarithmic phase bacterial suspension, and the final concentrations of 62.50, 31.25, and 15.63 μg/mL were achieved. After incubation at 37 °C for 3 h, bacterial cells were collected, washed, and treated with 0.6 mL of ice-cold lysis buffer to extract ATP. The bacterial solution was centrifuged at 4500 rpm for 10 min. The top layer was collected and frozen to prevent ATP loss. The supernatant and the same amount of ATP were mixed in a black opaque 96-well microtitration plate and measured using the multifunctional microplate detector (Tecan spark, Tecan, Männedorf, Switzerland).

### 2.10. Cell Cycle

*S. aureus* and *MRSA* in the logarithmic phase were treated with BBR, TA, BBR/TA MIX, and BBR-TA NPs at 37 °C for 3 h. The cells were then centrifugated at 4 °C and 5000 rpm for 5–10 min. The precipitates were collected, and bacteria cells were resuscitated with pre-cooled 1× PBS (4 °C). This procedure was repeated twice. Bacterial cells were fixed overnight with 70% ethanol at 4 °C, re-suspended, centrifuged, and washed with PBS. PBS was removed, and 500 μL of staining buffer, 25 μL of PI staining solution, and 10 μL of RNase were added and mixed. The mixture was incubated at 37 °C for 30 min without any exposure to light. The cell cycle was examined using flow cytometry (Novocyte, ACEA Biosciences Inc., San Diego, CA, USA).

### 2.11. Visual Assay of the Interaction Force between Bacteria and BBR-TA NPs

*S. aureus* and *MRSA* in the logarithmic phase were treated with BBR, TA, BBR/TA MIX, BBR-TA NPs, BP, and Cip for 3 h at 37 °C. Then, they were centrifuged at 4 °C for 5–10 min and washed with PBS three times. The bacteria cells were fixed with 2.5% glutaraldehyde for 4 h and dehydrated with increasing ethanol concentrations (30%, 50%, 70%, 80%, 90%, 95%, and 100%) for 10 min. After lyophilization and dehydration, the interaction between bacteria cells and BBR-TA NPs was observed using SEM.

### 2.12. Cytotoxicity Evaluation

BBR, TA, and BBR-TA NPs were evaluated to determine their cytotoxicity. HK-2 cell suspension was added to 96-well plates at an approximate density of 8 × 10^3^ cells/well and then incubated at 37 °C for 24 h. The original culture medium was discarded, and different drug concentrations were prepared with DMEM/F12 medium containing no fetal bovine serum as the treatment group. DMEM/F12 medium without fetal bovine serum was directly added as the control group. Medium without fetal bovine serum contains less components, which can reduce background interference and improve the accuracy of the experiment. Cell viability was detected using the CCK-8 kit (C0038, Beyotime, Shanghai, China), and the absorbance of each well was measured using the multifunctional microplate detector at 450 nm.

### 2.13. In Vivo Therapeutic Efficacy in Bacteria-Infected Wound Mice Model

The anti-infection and wound-healing effects of BBR, TA, BBR/TA MIX, BBR-TA NPs, BP, and Cip were evaluated using a mouse infection model. Male C57/6J mice (weight: 22–24 g) were purchased from Beijing Huafukang Biology Science and Technology Co., Ltd. (Beijing China). The mice were injected intraperitoneally with 4% chloral hydrate at a dose of 10 mg/kg. The hair on the back skin of the mice was shaved. The mice were sterilized with 0.5% iodophor, and an aseptic operation was performed. To establish an infectious wound model in mice, a full-thickness skin wound of 8 mm diameter was made by cutting the dorsal skin of mice with a sterile circular skin puncture device [40,41]. *MRSA* suspension was dropped directly onto the wound. The mice were randomly divided into control, BBR, TA, BBR/TA MIX, BBR-TA NPs, BP, and Cip groups. The concentration in each group was 15.63 μg/mL. Then, 20 μL of the drug was smeared on the wound surface of the mice, and the wound was bandaged with an aseptic gauze (administration occurred once every other day). Images of the wound healing process were taken by using a digital camera at different time points (Day 0, 3, 7, 11, and 15), and the wound area was quantified using image analysis software (ImageJ, National Institutes of Health, Bethesda, MA, USA). The wound healing rate was calculated using the following formula:

Relative wound area (%) = A_t_/A × 100% (2)

where A is the wound area immediately after surgery, and A_t_ is the wound area on days 3, 7, 11, and 15 after surgery.

### 2.14. Statistical Analysis

The obtained data were analyzed and presented as mean ± standard deviations (SDs) of *n* ≥ 3. For data analysis, we mainly use origin (2021) and Graphpad Prism 8 software (8.2.263, Graphpad software, San Diego, CA, USA). ImageJ software (V 1.8.0.112, National Institutes of Health, Bethesda, MD, USA) was used to quantify the wound area of mice. Statistical analyses of various parameters were performed using a *t*-test. *p* values of <0.05 were considered statistically significant.

## 3. Results and Discussion

### 3.1. Preparation and Characterization of BBR-TA NPs

**Figure 1:**
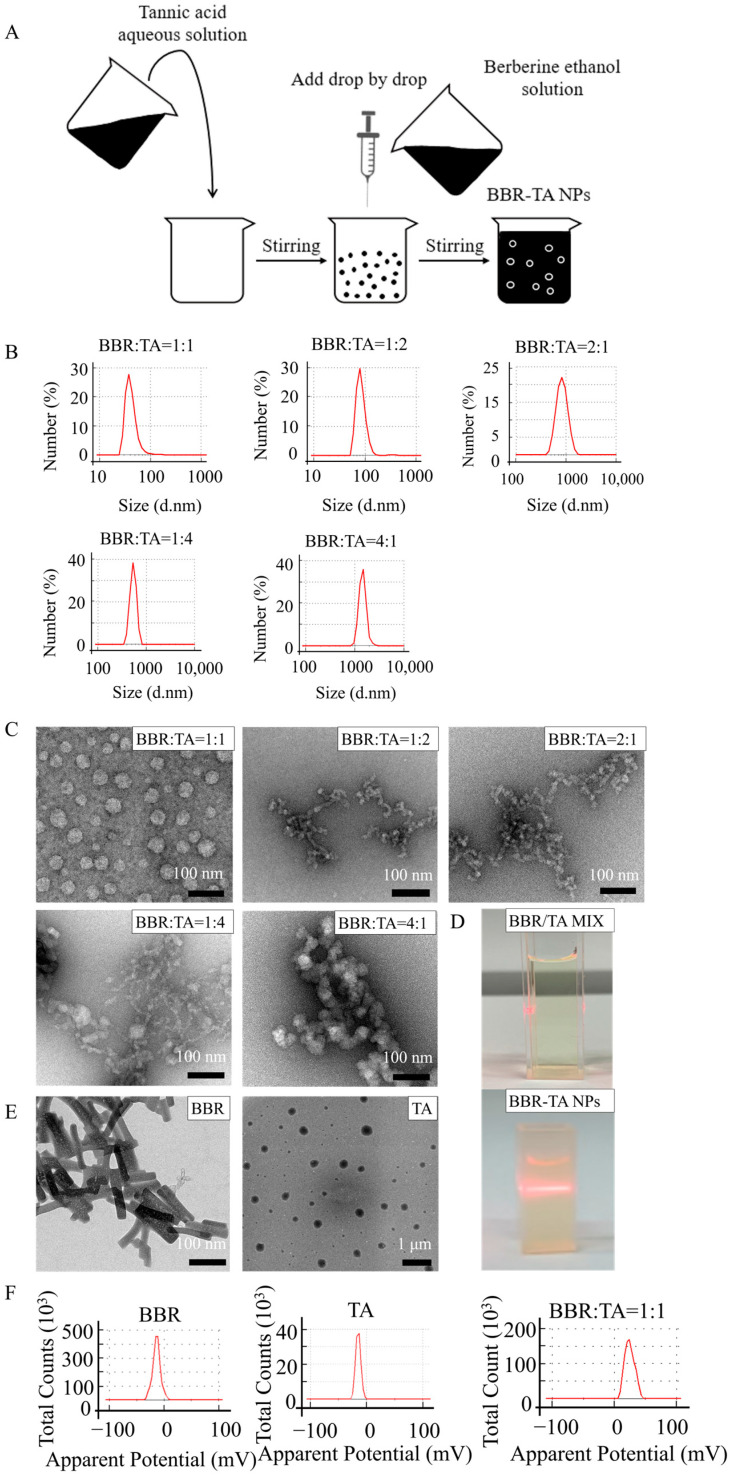
The characterization of BBR-TA NPs and their single components. (**A**) The schematic diagram of BBR-TA NP preparation process. (**B**) Particle size diagrams of nanoparticles composed of different proportions of BBR and TA. (**C**) Transmission electron microscopy diagrams of nanoparticles composed of BBR and TA in different proportions. (**D**) Tyndall effect diagrams of BBR/TA MIX and BBR-TA NPs (the molar ratio of BBR to TA is 1:1). (**E**) Transmission electron microscopy images of crude BBR and TA. (**F**) Zeta potential diagrams of BBR, TA, and BBR-TA NPs (the molar ratio of BBR to TA is 1:1).

Based on the BBR and TA structures, we speculated that BBR and TA co-assembled into carrier-free, biodegradable, safe, low-cost NPs without the participation of excipients. Figure 1A shows the preparation process of BBR-TA NPs. To investigate the co-assembly behavior of BBR and TA, different molar ratios (1:1, 1:2, 2:1, 1:4, and 4:1) of BBR and TA were added to deionized water. Figure 1B presents the results of the particle sizes. The morphology of each sample was visualized using TEM (Figure 1C). When the molar ratio was 1:1, the particle size was the smallest, and the PDI was 0.197, which indicated narrow size distribution and high dispersivity. When the molar ratio was 1:2, the particle size increased slightly. When the molar ratios were 1:4, 2:1, and 4:1, the particle size increased sharply. One study demonstrated that antibacterial activity increased with a decrease in the size of NPs [42]. Therefore, BBR-TA NPs at the 1:1 ratio were investigated in the follow-up study. The Tyndall effect of the BBR-TA NPs (1:1) was obvious compared with that of the physical mixture of BBR and TA (BBR/TA MIX) (Figure 1D). This reflects the successful co-assembly of BBR and TA into NPs [43,44]. The particle size of BBR-TA NPs (1:1) was approximately 42.50 nm (Figure 1B), and their zeta potential was +24.7 mV (Figure 1F). This was consistent with the TEM result of the BBR-TA NPs (Figure 1C). The TEM image (Figure 1E) showed that crude BBR was rod-shaped, while TA was spherical with a size of approximately 300 nm. Following co-assembly, spherical-shaped NPs with a good dispersion capability and a size of <100 nm were formed. Together, at the optimized molar ratio (1:1), spherically co-assembled NPs with a uniform size and a suitable polydispersity were obtained. Figure S1 shows that the particle size of BBR-TA NPs (*n*:*n* = 1:1) increases slightly with time, but the effect is not significant and tends to stabilize around the fifth day.

### 3.2. Co-Assembly Mechanism of BBR-TA NPs

**Figure 2:**
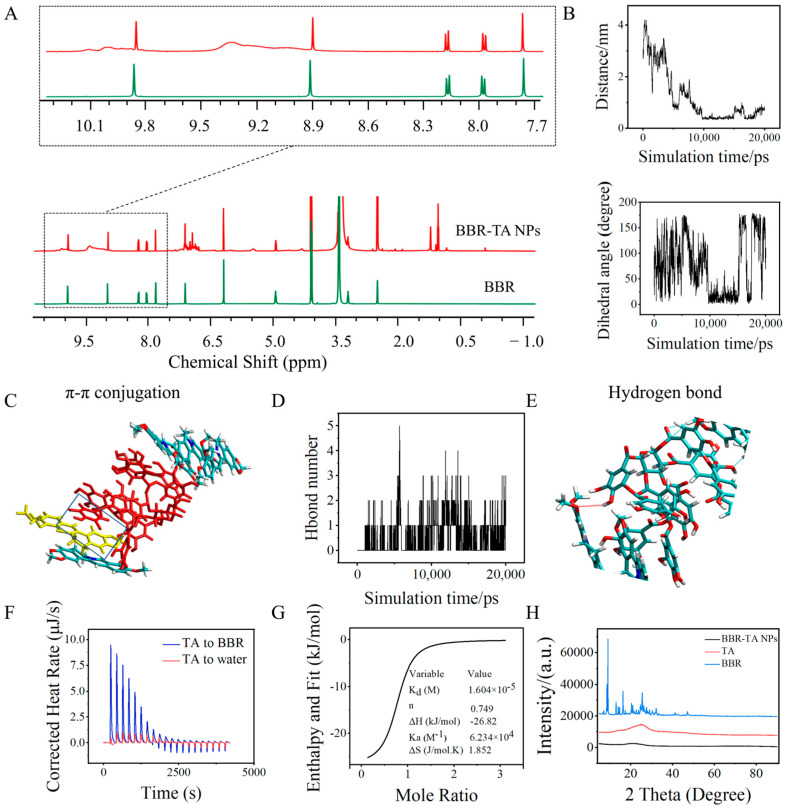
Co-assembly mechanism of BBR-TA NPs. (**A**) ^1^H NMR spectrum of BBR and BBR-TA NPs. (**B**–**E**) MD simulation of the interaction of BBR and TA. (**F**,**G**) ITC thermodynamic parameters of the titration of BBR-TA NPs by the BBR solution titrated with the TA solution (4 mM TA titration to 0.4 mM BBR; 4 mM TA titration to water). (**H**) X-ray diffractogram of BBR, TA, and BBR-TA NPs.

The ^1^H NMR test of the BBR-TA NPs and their single components was performed, and the results obtained were compared with those in dimethyl sulfoxide-d_6_ (DMSO-d_6_) to further clarify the interactions within the co-assembled NPs. The ^1^H NMR spectra of BBR-TA NPs (Figure 2A) presented H signals of BBR and TA, indicating that conjugated NPs were successfully synthesized. The chemical shift of the H signal of the isoquinoline ring in BBR was observed to be from 9.884, 8.935, and 8.003 ppm to 9.872, 8.921, and 7.997 ppm, that is, from the lower signal field to the upper signal field. Table S1 presents the specific data. Owing to the large structural formula of TA and the unclear results of the hydrogen spectrum analysis, we speculated that the isoquinoline ring of BBR and the benzene ring of TA form a π-π stacking structure because of the change in the hydrogen signal of the BBR isoquinoline ring.

To validate our speculation, BBR and TA were further subjected to MD simulations using the molecular docking software Gromacs. In the BBR and TA simulation system, two rings produced conjugation, and their distance and dihedral angle varied with time (Figure 2B). At approximately 10,000 ps (10 ns), the distance was approximately 0.4 nm, and the dihedral angle of the two ring planes was close to 0° to form a conjugate structure. Figure 2C presents the formation of π-π conjugation between BBR and TA. Figure 2D shows the change in the number of hydrogen bonds of BBR-TA NPs with an increase in simulation time. Figure 2E presents the hydrogen bond configuration, which confirms the hydrogen bond interaction between BBR and TA. Overall, BBR molecules were predicted to co-assemble with TA molecules. The final structure was likely to be held together by a synergetic supramolecular interaction, including π-π stacking and hydrogen bond interactions. Detailed molecular simulation data of the BBR–TA interaction are given in Figures S2 and S3. The predictions of these MD simulations are consistent with our experimental data and strongly support the hypothesis that BBR and TA molecules are co-assembled into BBR-TA NPs.

The thermodynamic mechanism of the BBR–TA interaction was studied via isothermal titration calorimetry (ITC). Figure 2F presents the isothermal titration curves of BBR and TA. Two groups of experiments were also conducted in this part, including the titration of the TA solution into purified water and that of the TA solution into a BBR solution. The binding heat of purified water titrated by TA was set as the benchmark, and the titration of TA into BBR was performed in the test group. Table S2 lists all the energy changes in the interaction, as determined via ITC. The heating curve of the titration process was fitted with the Nano Analyze software. By fitting the titration curve, we obtained the combined thermodynamic parameters of BBR and TA. The specific data are presented in Table S3. According to the Wiseman isotherm theory, the S-shaped fitting curve represents strong affinity, whereas the parabola represents low affinity. Thus, the S-shaped fitting curve (Figure 2G) indicates that BBR has a strong affinity for TA [45]. The negative ΔH value supported that the BBR–TA binding was an enthalpy-driven reaction. The negative Gibbs energy change (ΔG) indicates that the reaction of BBR and TA is a spontaneous process.

The analysis of the potential internal kinetics of drugs via X-ray diffractometry is beneficial for studying the co-assembly mechanism. The XRD patterns of BBR exhibited sharp and strong diffraction peaks at 2θ, which were 8.65°, 9.16°, 16.36°, 25.54°, 27.62°, and 47.11°, and indicated that BBR was crystallized. BBR-TA NPs (Figure 2H) formed broad peaks, indicating a change in the crystallization properties of the components. Amorphous materials have higher free energy than their corresponding crystalline forms. Compared with their respective crystal forms, dissolving drugs with less crystallization or in the amorphous phase is easier, and they have a higher dissolution rate. Therefore, modifying crystalline properties by preparing drugs into NPs further improves bioavailability [46,47].

On combining a series of characterization analyses, including the ^1^H NMR analysis, MD simulation, ITC, and XRD analysis of BBR-TA NPs and their single components, BBR and TA were found to spontaneously co-assemble into NPs via hydrogen bond and π-π stacking interactions.

### 3.3. Antibacterial Effect of BBR-TA NPs

**Figure 3:**
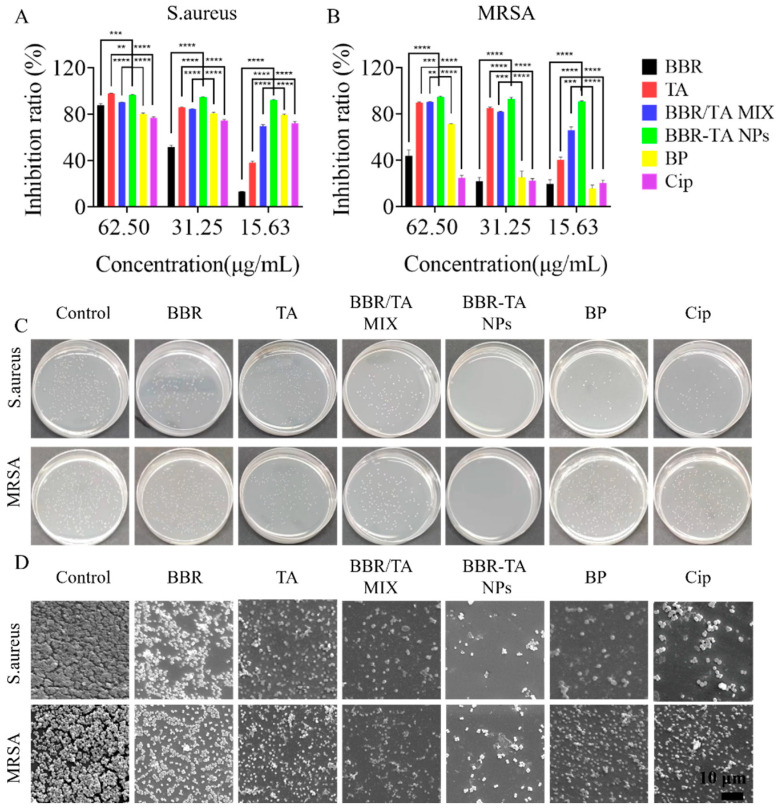
Antibacterial effect of BBR-TA NPs. (**A**) Inhibition ratio of *S. aureus* treated with different concentrations of BBR, TA, BBR/TA MIX, BBR-TA NPs, BP, and Cip. (**B**) The inhibition ratio of *MRSA* was treated with different concentrations of BBR, TA, BBR/TA MIX, BBR-TA NPs, BP, and Cip. (**C**) The survival numbers of *S. aureus* and *MRSA* were treated with blank media (control), BBR, TA, BBR/TA MIX, BBR-TA NPs, BP, and Cip. (**D**) SEM images of biofilm incubated with the blank media (control), BBR, TA, BBR/TA MIX, BBR-TA NPs, BP, and Cip against *S. aureus* and *MRSA* (scale bar = 10 μm). ** *p* < 0.01; *** *p* < 0.001; **** *p* < 0.0001.

In the in vitro antibacterial experiment conducted using fluorescence staining, we studied the antibacterial activity of BBR-TA NPs against *S. aureus* and *MRSA*. Syto9 (a green fluorescent dye) and propidium iodide (PI, a red fluorescent dye) are used to study various bacterial species from various environments. Although a good linear relationship exists between green/red fluorescence and living cells, considering that some nucleic acids of damaged bacteria are lost during centrifugation or degradation, the green fluorescence value and the corresponding percentage of living bacteria are used to calculate bacterial survival and inhibition rates [48,49]. Figure 3A presents the inhibition ratio of *S. aureus* treated with different drugs. For *S. aureus*, the minimum inhibitory concentration of BBR was 62.50 μg/mL, and the antibacterial rate of BBR-TA NPs at 62.50 μg/mL was 96.64% ± 0.29%. As the concentration decreased, the antibacterial effect of the BBR-TA NPs became better than that of crude BBR. At 15.63 μg/mL, the antibacterial rates of BBR and the BBR-TA NPs were 13.39% ± 0.17% and 90.32% ± 0.36%, respectively. The antibacterial rates of BP and Cip on *S. aureus* were basically between 70% and 80%. BBR-TA NPs exhibited better antibacterial activity than the two first-line antibiotics. Figure 3B presents the inhibition rate of *MRSA* treated with different drugs. For *MRSA*, the inhibition ratios of BBR and BBR-TA NPs were 43.90% ± 5.01% and 94.61% ± 0.83%, respectively, at 62.50 μg/mL. As the concentration decreased, the antibacterial rates of BBR and the BBR-TA NPs were 19.69% ± 3.53% and 90.66% ± 0.63%, respectively, at 15.63 μg/mL. First-line antibiotics, such as BP and Cip, exhibited a good antibacterial effect on *S. aureus*, but their antibacterial effect on *MRSA* was poor. The BBR-TA NPs exhibited a good antibacterial effect on *S. aureus* (Table S4) and *MRSA* (Table S5) at a range of concentrations. All samples exhibited dose-dependent inhibition on bacterial proliferation.

The antibacterial effect of the BBR-TA NPs on *S. aureus* and *MRSA* was determined using the spread plate method. *S. aureus* and *MRSA* were treated with all samples. The results (Figure 3C) are consistent with those obtained by fluorescence staining.

With the in-depth understanding of some chronic and intractable diseases caused by common bacteria in some environments, the main obstacle in treating bacterial infectious diseases is biofilm [50]. The scavenging ability of the BBR-TA NPs against bacterial biofilm was observed using SEM. A dense biofilm was formed on both *S. aureus* and *MRSA* of the control group, and a few dead bacteria were observed (Figure 3D). BBR treatment made the biofilm thinner and looser, while the BBR-TA NP treatment disintegrated a large amount of biofilm, thereby exposing smooth cellular creeps.

The literature shows that nanoparticles formed by berberine, rhein [37], and cinnamic acid [44] can also improve the antibacterial effect. Among them, berberine–flavonoid glycoside nanoparticles [51] have a better antibacterial effect than the first-line antibiotics norfloxacin, amoxicillin, and ciprofloxacin. At the concentration of 15.63 μg/mL, the antibacterial rate of the BBR-TA NPs was better than that of berberine–rhein nanoparticles and berberine–cinnamic acid nanoparticles. The BBR-TA NPs prepared by us have a good antibacterial effect on *S. aureus* and *MRSA*, indicating that the particle size of BBR decreases after the formation of nanoparticles and increases the contact area with bacteria, resulting in bacterial death.

### 3.4. Antibacterial Mechanism of BBR-TA NPs

**Figure 4:**
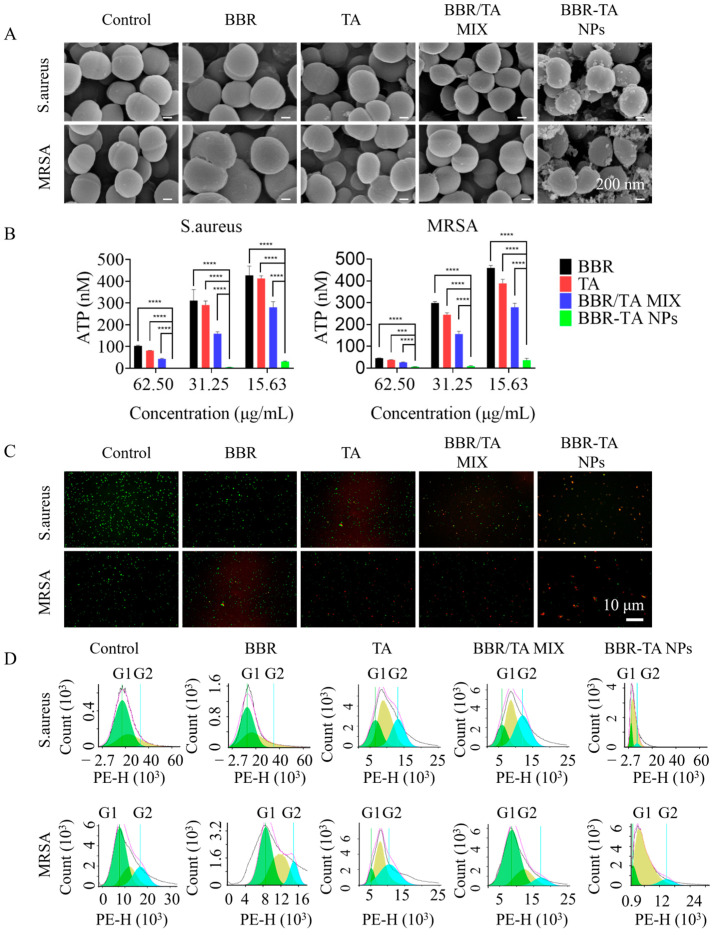
Antibacterial mechanism of BBR-TA NPs. (**A**) Interaction of BBR, TA, BBR/TA MIX, and BBR-TA NPs with *S. aureus* and *MRSA* via SEM (scale bar = 200 nm). (**B**) The effect of BBR, TA, BBR/TA MIX, and BBR-TA NPs on the membrane integrity of *S. aureus* and *MRSA* cells via LIVE/DEAD fixable dead cell stain assay (scale bar = 10 μm). (**C**) The effect of BBR, TA, BBR/TA MIX, and BBR-TA NPs on the concentration of ATP in *S. aureus* cells and *MRSA* cells. (**D**) The effect of BBR, TA, BBR/TA MIX, and BBR-TA NPs on the cell cycle distribution of *S. aureus* and *MRSA*. *** *p* < 0.001; **** *p* < 0.0001.

To further explain the antibacterial behavior of the BBR-TA NPs, the interaction between NPs and bacteria as well as bacterial morphology were investigated using SEM. After *S. aureus* and *MRSA* were treated with the BBR-TA NPs, the BBR-TA NPs were found to uniformly adhere to the surface of the bacteria (Figure 4A). However, the bacteria treated with BBR, TA, and BBR/TA MIX had no adhesive interaction with the bacterial surface. Zeta potential, along with particle size and chemistry, is a highly relevant parameter controlling antimicrobial effects. The zeta potential (Figure 1E) of the BBR, TA, and BBR-TA NP aqueous solutions were −13.70 mV, −14.80 mV, and +24.70 mV, respectively. Previous studies have revealed that positively charged nanoparticles interact with a negatively charged bacterial surface via electrostatic action, which provides a reasonable explanation for the adhesion of nanoparticles to the bacterial surface [44,52,53]. Moreover, the strongly positive zeta potential of BBR-TA NPs promotes the interaction of nanoparticles with the cell membrane, leading to membrane disruption and a reduction in viability. Normal *S. aureus* and *MRSA* cells are round with smooth surfaces. No significant difference was observed between the BBR and control groups. With the BBR-TA NPs, *S. aureus* and *MRSA* cells were no longer smooth and became dry and then shrunk.

ATP is a type of high-energy phosphate compound. It is a substance critical for biological growth, replication, and survival. In cells, ATP is a kind of high-energy phosphate compound. In cells, it can transform with ADP to achieve energy storage and release, thus ensuring the energy supply of various life activities of the cell. ATP is the most direct energy source in organisms [54]. In this study, after the treatment of *S. aureus* and *MRSA* with the BBR-TA NPs (Figure 4B), the intracellular ATP concentration decreased significantly. The decrease in ATP concentration may be due to the damage to the bacterial cell membrane, which results in the intracellular ATP leakage acceleration of the hydrolysis rate by intracellular proton pump ATPase [49].

*S. aureus* and *MRSA* were stained with SYOT9 and PI. The green fluorescent stain labeled live bacterial cells, whereas the red fluorescent stain labeled dead bacterial cells. Most bacteria (*S. aureus* and *MRSA*) in the control group exhibited green fluorescence under the fluorescence microscope, indicating that the cell membrane integrity was maintained (Figure 4C). However, after treatment with the same concentration of BBR, TA, BBR/TA MIX, and the BBR-TA NPs, the green fluorescence in bacterial cells decreased and red fluorescence appeared, which was consistent with the results obtained using micro-tablet readers. The BBR-TA NPs destroyed the bacterial cell membrane structure and promoted the outflow of nucleic acid. Compared with the control group, the BBR-TA NP group exhibited a decrease in the total number of bacteria and an inhibition of bacterial proliferation, which was indicated by the significantly increased red fluorescence. This also validates the speculation of ATP leakage.

Bacterial proliferation is regulated by the cell cycle process. The cell cycle is divided into different stages, namely, I, R, and D phases, which are the same as the Gap 1 (G1) phase, the Gap 2 (G2) phase, and the Synthesis (S) phase in eukaryotic cells [49]. Controlling the cell cycle process may act as an effective strategy for controlling cell growth. As shown in Figure 4D, the treatment of *S. aureus* and *MRSA* with BBR-TA NPs blocks the cell cycle and makes the cell cycle stagnant in the S phase. This indicated that the BBR-TA NPs inhibit DNA replication, thereby blocking bacterial cell division and affecting bacterial proliferation.

### 3.5. Therapeutic Effect of BBR-TA NPs on MRSA-Infected Wounds in Mice

**Figure 5:**
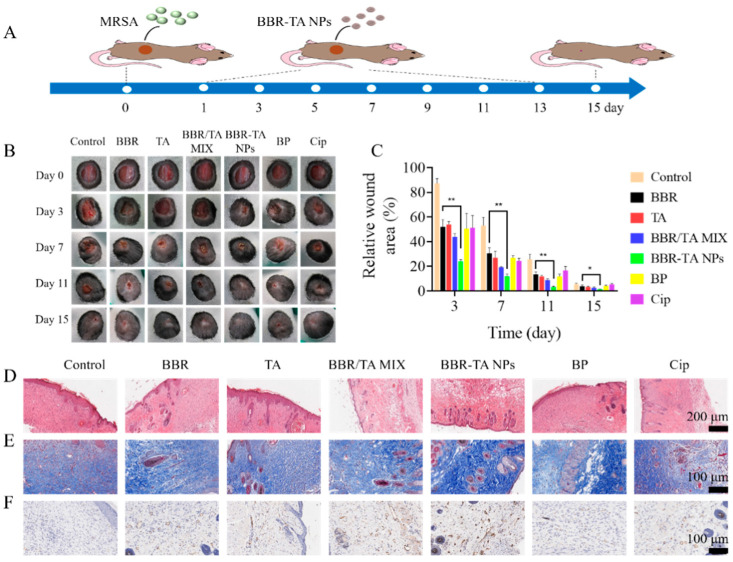
Treatment efficiency of *MRSA* bacteria-infected wound in mice with BBR-TA NPs. (**A**) Schematic diagram of the construction of mice model of wound infection and treatment process. (**B**) Representative images of the cutaneous wound in each group on days 0, 3, 7, 11, and 15 after surgery. (**C**) The relative wound area in each group at different time points. * *p* < 0.05; ** *p* < 0.01. (**D**) Histological analysis of wound tissues by H&E on day 15 after surgery (scale bar = 200 μm). (**E**) Histological analysis of wound tissues by Masson’s trichrome staining on day 15 after surgery (scale bar = 100 μm). (**F**) IHC image of anti-CD31 regenerated wound tissues on day 15 after surgery (scale bar = 100 μm).

The aforementioned in vitro experiments systematically proved that the BBR-TA NPs exhibited good antibacterial properties against *MRSA*. Therefore, the mouse model of skin wound infections was used to evaluate the therapeutic effect of the BBR-TA NPs in vivo. As the most common pathogen causing skin and soft tissue infection, *MRSA* was selected as the object of this in vivo study. A full-thickness skin wound was created on the back of mice, and then, this wound was infected with *MRSA*. After 24 h, 20 μL of PBS (control group), BBR, TA, BBR/TA MIX, BBR-TA NPs, BP, and Cip was smeared on the wound in each group to determine the antibacterial activities and wound-healing capacities of these compounds in vivo. Figure 5A is a schematic of the whole experimental process of wound healing. The wound photos, recorded at different time points, are presented in Figure 5B. The BBR-TA NP group exhibited significantly faster healing than the other groups at all time points. On the 11th day, the wound surface in the BBR-TA NP group basically healed and was covered with a new epithelium. In contrast, the wound in the other groups was open and exhibited eschar formation. The wound scabs gradually fell off, and new tissue regenerated. The column chart in Figure 5C presents a clear change in wound size in different groups on days 3, 7, 11, and 15. As expected, the calculated relative wound area confirmed that the BBR-TA NP group promoted healing at all time points and was superior to other groups.

To test the wound healing mechanism, we used the tissue of newborn mouse skin for hematoxylin and eosin (H&E) staining, Masson’s trichrome staining, and CD31 immunohistochemical staining. The result of H&E staining is shown in Figure 5D. The control group was not completely re-epithelialized and had more inflammatory cell infiltration. In contrast, the Cip group exhibited complete re-epithelialization and the regeneration of some skin appendages, such as sebaceous glands, but more inflammatory cell infiltration was still observed. The BP group exhibited the regeneration of skin appendages, such as hair follicles and sebaceous glands, but the skin was not completely re-epithelialized, and more inflammatory cell infiltration was observed. In the BBR group and the TA group, the wound was re-epithelialized and exhibited the regeneration of skin appendages. The BBR/TA MIX group showed less inflammatory cell infiltration. The BBR-TA NP group exhibited the best wound healing efficacy. This group also exhibited the complete regeneration of the skin appendages and complete re-epithelialization, thereby exhibiting the largest number of hair follicles. Meanwhile, inflammatory cell infiltration was not observed. Altogether, the aforementioned results suggest that, with BBR-TA NP treatment, the healing process can be remarkably accelerated within a short period.

Masson’s trichrome staining was performed to evaluate collagen density in the regenerated tissue on day 15. The collagen density was considerably increased in all groups (Figure 5E). In the control group, blue collagen fibers could be seen in the wound skin, collagen staining was weak, and disordered muscle fibers could be observed. Compared with the other groups, the BBR-TA NP group exhibited the highest density of collagen. The results indicated that BBR-TA NPs could remarkably promote collagen deposition and accelerate wound healing [55,56].

CD31, also known as platelet-endothelial cell adhesion molecule-1, belongs to the immunoglobulin superfamily. It has a crucial role in eliminating aging neutrophils in vivo. In immunohistochemistry, CD31 is mainly used to prove the presence of endothelial cells and evaluate angiogenesis. Regarding CD31 immunohistochemistry staining in newborn tissues, compared with the control group, other treatment groups presented a strong positive staining signal of CD31. The BBR-TA NP group exhibited the highest level of angiogenesis compared with the other groups, which further facilitated the healing process in vivo (Figure 5F).

### 3.6. Biocompatibility Evaluation of BBR-TA NPs

**Figure 6:**
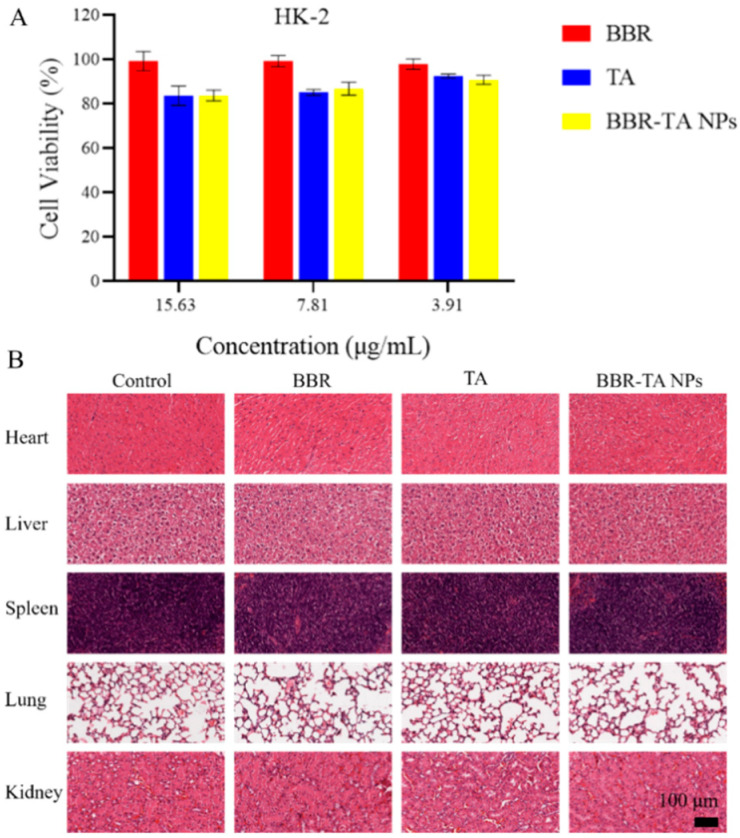
Biosafety evaluation of BBR-TA NPs. (**A**) Cell viability after incubation with BBR, TA, and BBR-TA NPs for treatment of 24 h. (**B**) Representative H&E staining photomicrographs of the major organs of the mice including the heart, liver, spleen, lung, and kidney at day 15 after surgery (scale bar = 100 μm).

To confirm the biosafety of BBR-TA NPs, the in vitro cytotoxicity test was conducted, and an in vivo mouse model was established. HK-2 cells were used to study the cytotoxicity of BBR, TA, and the BBR-TA NPs. The BBR-TA NPs were slightly more cytotoxic than BBR (concentration). However, when the concentration of the BBR-TA NPs was 15.63 μg/mL, their inhibition rate on *S. aureus* and *MRSA* was >80%, whereas that on HK-2 cells was <20%, which indicates that the BBR-TA NPs have no obvious cytotoxicity (Figure 6A) [51].

Any drug application in disease treatment is associated with the most basic problem of biosafety. In the whole mouse wound model experiment, no death or abnormal behavior was observed in all mice treatment groups compared with the control group. Histological results revealed the microstructure of the main organs of mice, including the heart, liver, spleen, lung, and kidney (Figure 6B). No microscopic difference or abnormality in the organs was observed between the treatment and control groups. The results are consistent with those of the cytotoxicity test, and the nanodrug-mediated combination therapy was considered safe and does not involve any activation of the inflammation response. Therefore, BBR-TA NPs are a promising nano-antibacterial agent for the clinical treatment of wound infections.

## 4. Conclusions

In summary, BBR was co-assembled with TA into carrier-free BBR-TA NPs by using a simple “green” preparation procedure. BBR and TA molecules were co-assembled via π−π stacking interactions and hydrogen bonds to form nanostructures. They exhibited a robust therapeutic effect because of their higher adherence to bacteria and penetration into bacteria, leading to a synergetic converging attack against *MRSA*. Even if the concentration of the BBR-TA NPs was as low as 15.63 μg/mL, the antibacterial rate against *S. aureus* and *MRSA* was more than 80%. Furthermore, the BBR-TA NPs exhibited an enhanced healing efficacy against *MRSA*-infected wounds in the mouse model. The use of this co-assembly strategy in the antibacterial field could avoid the use of any carriers and accessories. Collectively, co-assemblies designed using active phytochemicals can offer improved clinical benefits with no obvious cytotoxicity, thereby promoting the clinical translation of nanomedicine. The self-assembly of small molecular compounds in plant drugs to form nanoparticles provides a new idea for solving the drug resistance characteristics of *MRSA* in clinics and is expected to solve the problem of bacterial resistance in the world.

## Data Availability

The data underlying this article will be shared on reasonable request to the corresponding author.

## Supplementary Materials

The following supporting information can be downloaded at: https://www.mdpi.com/article/10.3390/pharmaceutics15071782/s1, Figure S1: Stability of BBR-TA NPs measured by DLS; Figure S2: π-π conjugation of molecular simulation system of BBR and TA; Figure S3: Hydrogen bonding of molecular simulation system of BBR and TA; Table S1: ^1^H-NMR chemical shift of BBR in BBR-TA NPs self-assembly; Tables S2 and S3: Energy changes data for the interactions obtained from ITC; Table S4: Inhibition rates of different concentrations of BBR, BBR-TA NPs, BP and Cip on *S. aureus*; Table S5: Inhibition rates of different concentrations of BBR, BBR-TA NPs, BP and Cip on *MRSA*.
