# Correction: Zheng et al. Fabrication of Co-Assembly from Berberine and Tannic Acid for Multidrug-Resistant Bacteria Infection Treatment. *Pharmaceutics* 2023, *15*, 1782

**DOI:** 10.3390/pharmaceutics16111418

**Published:** 2024-11-05

**Authors:** Tingting Zheng, Huan Chen, Chenyang Wu, Jinrui Wang, Mengyao Cui, Hanyi Ye, Yifan Feng, Ying Li, Zhengqi Dong

**Affiliations:** 1 Drug Delivery Research Center, Institute of Medicinal Plant Development, Chinese Academy of Medical Sciences, Peking Union Medical College, Beijing 100193, China; s2020009014@student.pumc.edu.cn (T.Z.); 15030413919@163.com (H.C.); wuchenyang54@163.com (C.W.); 15776622452@139.com (J.W.); cuimengyao5521@163.com (M.C.); yehy6620@163.com (H.Y.); fyf5501@163.com (Y.F.); 2 Key Laboratory of Bioactive Substances and Resources Utilization of Chinese Herbal Medicine, Ministry of Education, Chinese Academy of Medical Sciences, Peking Union Medical College, Beijing 100094, China; 3 Key Laboratory of New Drug Discovery Based on Classic Chinese Medicine Prescription, Beijing 100700, China; 4 Beijing Key Laboratory of Innovative Drug Discovery of Traditional Chinese Medicine (Natural Medicine) and Translational Medicine, Beijing 100700, China

In the original publication [1], there was a mistake in Figure 4. In Figure 4C, *S. aureus*-BBR vs. MRSA-Control, *S. aureus*-TA vs. MRSA-BBR, and MRSA-TA vs. MRSA-BBR/TA MIX were repeated. All the pictures were combined using PPT software and then exported. Because the experimental steps for detecting the effects of the drugs on *S. aureus* and MRSA were exactly the same, and the experimental results of the above groups were similar, this led to an insertion error. However, the description of the results and the conclusion are correct. The corrected Figure 4 appears below. The order of the legend of Figure 4B,C is reversed, and the legend of Figure 4B should correspond to Figure 4C, and the legend of Figure 4C should correspond to Figure 4B. The correct legend appears below. The authors state that the scientific conclusions are unaffected. This correction was approved by the Academic Editor. The original publication has also been updated.

## Figures and Tables

**Figure 4:**
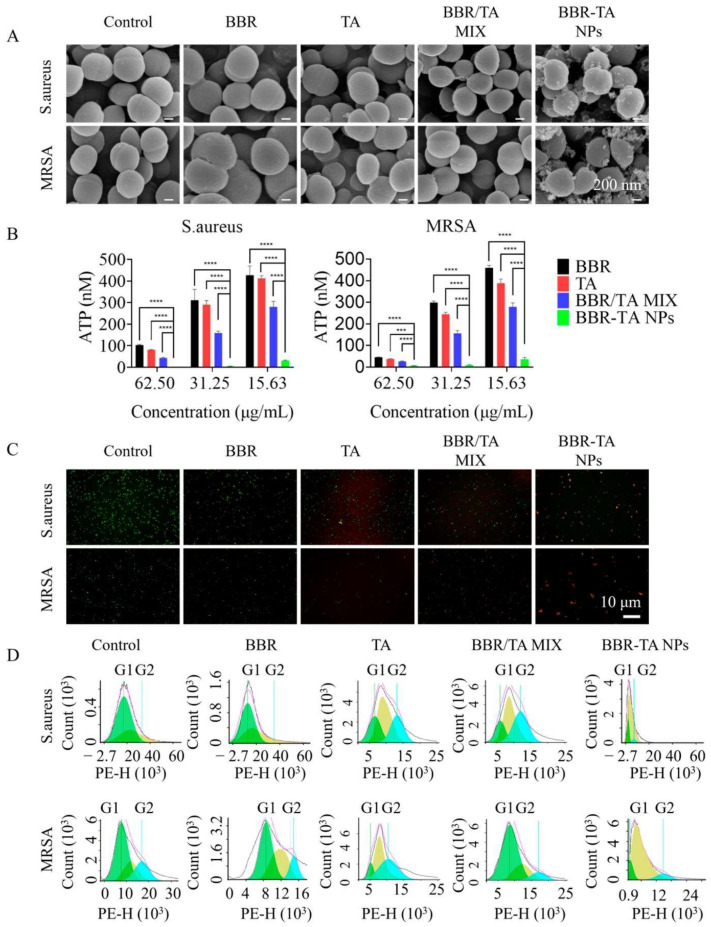
Antibacterial mechanism of BBR-TA NPs. (**A**) Interaction of BBR, TA, BBR/TA MIX, and BBR-TA NPs with *S. aureus* and MRSA via SEM (scale bar = 200 nm). (**B**) The effect of BBR, TA, BBR/TA MIX, and BBR-TA NPs on the concentration of ATP in *S. aureus* cells and MRSA cells. (**C**) The effect of BBR, TA, BBR/TA MIX, and BBR-TA NPs on the membrane integrity of *S. aureus* and MRSA cells via LIVE /DEAD fixable dead cell stain assay (scale bar = 10 μm). (**D**) The effect of BBR, TA, BBR/TA MIX, and BBR-TA NPs on the cell cycle distribution of *S. aureus* and MRSA. *** *p* < 0.001; **** *p* < 0.0001.
