# Supplementary material for: Fabrication of Co-Assembly from Berberine and Tannic Acid for Multidrug-Resistant Bacteria Infection Treatment
Source: Pharmaceutics. 2023 Jun 21;15(7):1782. doi: 10.3390/pharmaceutics15071782 (PMC10383063; doi:10.3390/pharmaceutics15071782)
Supplement: Supplementary file 1 [file pharmaceutics-15-01782-s001.zip › pharmaceutics-2410474-supplementary.pdf]

Fabrication of Co-Assembly from Berberine and Tannic Acid for Multidrug-Resistant Bacteria Infection Treatment

Tingting Zheng [1], Huan Chen [1], Chenyang Wu [1], Jinrui Wang [1], Mengyao Cui [1], Hanyi Ye [1], Yifan Feng [1], Ying Li [1,2,3,4,*] and Zhengqi Dong [1,2,3,4,*]

1 Drug Delivery Research Center, Institute of Medicinal Plant Development, Chinese Academy of Medical Sciences, Peking Union Medical College, Beijing 100193, China; s2020009014@student.pumc.edu.cn (T.Z.); 15030413919@163.com (H.C.); wuchenyang54@163.com (C.W.); 15776622452@139.com (J.W.); cuimengyao5521@163.com (M.C.); yehy6620@163.com (H.Y.); fyf5501@163.com (Y.F.)
2 Key Laboratory of Bioactive Substances and Resources Utilization of Chinese Herbal Medicine, Ministry of Education, Chinese Academy of Medical Sciences, Peking Union Medical College, Beijing 100094, China
3 Key Laboratory of New Drug Discovery Based on Classic Chinese Medicine Prescription, Beijing 100700, China
4 Beijing Key Laboratory of Innovative Drug Discovery of Traditional Chinese Medicine (Natural Medicine) and Translational Medicine, Beijing 100700, China
* Correspondence: yli@implad.ac.cn (Y.L.); zqdong@implad.ac.cn (Z.D.)

Particle Size Measurement: The freeze-dried BBR-TA NPs powder was uniformLy dispersed in water and placed in the sample cell of dynamic light scattering meter to measure their particle size and Zeta potential.

Transmission electron microscopic analysis of berberine and co-assembled nanoparticles: Drop the sample solution onto the carbon coated copper mesh (300 mesh). After adsorbing 0.5 min, the excess part is extracted with filter paper, and then the 20 min is placed statically. The sample was then further dried in vacuum for 12 hours. Electron microscope images were taken by transmission electron microscope at 200kV acceleration voltage.

Table S1. ^{1}H-NMR chemical shift of BBR in BBR-TA NPs self-assembly.

	BBR-TA NPs	BBR	Δδ		BBR-TA NPs	BBR	Δδ
H-5	9.872	9.884	-0.012	H-7	7.997	8.003	-0.006
H-6	8.921	8.935	-0.014	H-1	7.790	7.785	0.005
H-8	8.198	8.193	0.005				

X-ray diffraction: the X-ray diffraction patterns of untreated BBR and prepared BBR-TA NPs were recorded by X-ray diffractometer. The working voltage is 40 kV and the working current is 40mA. The starting angle 2θ is 5° and the end angle is 90°.

Molecular Dynamics Simulation： In this work, a standard molecular mechanics potential model was used with the following functional form:

$$u(r^N) = \sum_{bonds} \frac{k_i}{2}(l_i - l_{i,0})^2 + \sum_{angles} \frac{k_i}{2}(\theta_i - \theta_{i,0})^2 + \sum_{torsions} \frac{V_n}{2}(1 + \cos(n\omega - \gamma))$$

$$+ \sum_{i=1}^{N} \sum_{j=i+1}^{N} \left(4\varepsilon_{ij} \left[\left(\frac{\sigma_{ij}}{r_{ij}} \right)^{12} - \left(\frac{\sigma_{ij}}{r_{ij}} \right)^{6} \right] + \frac{q_i q_j}{r_{ij}} \right)$$

where the first three terms are the bonded interactions, including bond, angle, and torsion interactions, and the second terms are nonbonded interactions, including van der Waals (VDW) and Coulombic interactions. For different kinds of atoms, the Lorentz-Berthelot mix rules were adopted for vdW interactions, which is following the equation:

$$\sigma_{ij} = \frac{1}{2}(\sigma_{ii} + \sigma_{jj});\ \varepsilon_{ij} = (\varepsilon_{ii} * \varepsilon_{jj})^{1/2}$$

According to the experimental conditions, the system were constructed. The initial configurations of the systems were constructed using the Packmol software. The simulations were performed using GROMACS package (version 2019.3) with the all-atom OPLS (optimized performance for liquid systems) force field. For the system, the steep descent method was used to minimize the energy of the system. Then, molecular dynamics simulations under NPT ensemble at 298 K and 1 atm were performed for 20 ns for the system to collect trajectories for the subsequent data analysis. LINCS algorithm was applied to constrain the bond lengths of other components. Periodic boundary conditions were applied in all three directions. The temperature was maintained using the V-rescale thermostat algorithm. The cut-off distance for the Lennard-Jones and electrostatic interactions was 1.2 nm. Particle mesh Ewald method

was used to calculate the long-range electrostatic interactions. Configurations were visualized using Visual Molecular Dynamics software.

Bacterial resuscitation and preparation of bacterial reserve solution: 1.3 g of the medium nutritious gravy and broth medium was dissolved in 100 mL of purified water, bacteria were dispersed in the liquid medium nutritious gravy and broth, 37 ^{o}C and 200 rpm were rotated and cultured overnight, and bacteria were cultured to logarithmic phase for subsequent bacteriostatic experiments.

Biofilm culture: *Staphylococcus aureus* and *multidrug resistant Staphylococcus aureus* were dispersed in the liquid medium of nutritious gravy and broth. After the bacteria were cultured in the logarithmic phase, the bacterial suspension was diluted to 1×10^{7} CFU/mL using the medium containing 10% glucose. The biofilm was cultured on the cell climbing table and the biofilm was formed 24 hours later.

Table S2. Energy changes data for the interactions obtained from ITC.

Number	TA to BBR (μJ)	TA to H2O (μJ)	Correction (μJ)
1	-329	4.896	-333.896
2	-298.4	-42.34	-256.06
3	-266.2	-40.34	-225.86
4	-229.5	-38.06	-191.44
5	-189.6	-36.63	-152.97
6	-150	-34.74	-115.26
7	-106.6	-31.34	-75.26
8	-68.61	-26.91	-41.7
9	-40.88	-21.06	-19.82
10	-20.81	-15.43	-5.38
11	-5.942	-9.566	3.624
12	1.009	-4.226	5.235
13	6.656	-1.633	8.289
14	9.313	2.506	6.807
15	11.03	5.06	5.97
16	12.37	6.069	6.301
17	12.53	6.814	5.716
18	11.88	7.913	3.967
19	12.43	7.722	4.708
20	12.18	8.992	3.188

Table S3. Energy changes data for the interactions obtained from ITC.

NO.	Ka（L/mol）	ΔG(KJ/mol)	ΔH（KJ/mol）	-TΔS（KJ/mol）
TA to BBR	6.234×10^{4}	-26.3	-26.82	-0.552

Table S4. Inhibition rates of different concentrations of BBR, BBR-TA NPs, BP and Cip on *S.aureus*.

**Concentration**	**BBR**	**BBR-TA NPs**	**BP**	**Cip**
62.50 μg/mL	87.91%±1.59%	96.64%±0.29%	80.73%±0.90%	76.86%±0.74%
31.25 μg/mL	51.54%±1.63%	94.61%±0.47%	80.05%±1.03%	74.60%±0.86%
15.63 μg/mL	13.39%±0.17%	90.32%±0.36%	79.46%±0.53%	72.10%±1.50%

Table S5. Inhibition rates of different concentrations of BBR, BBR-TA NPs, BP and Cip on *MRSA*.

**Concentration**	**BBR**	**BBR-TA NPs**	**BP**	**Cip**
62.50 μg/mL	43.90%±5.01%	94.61%±0.83%	40.65%±1.83%	23.58%±1.72%
31.25 μg/mL	21.93%±3.25%	92.74%±1.58%	25.32%±5.31%	22.36%±1.90%
15.63 μg/mL	19.69%±3.53%	90.66%±0.63%	15.66%±2.97%	20.28%±2.38%

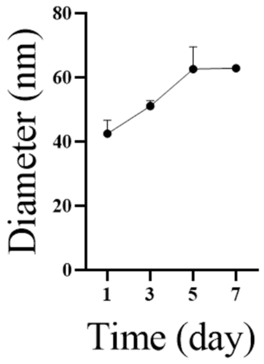

Figure S1. Stability of BBR-TA NPs measured by DLS.

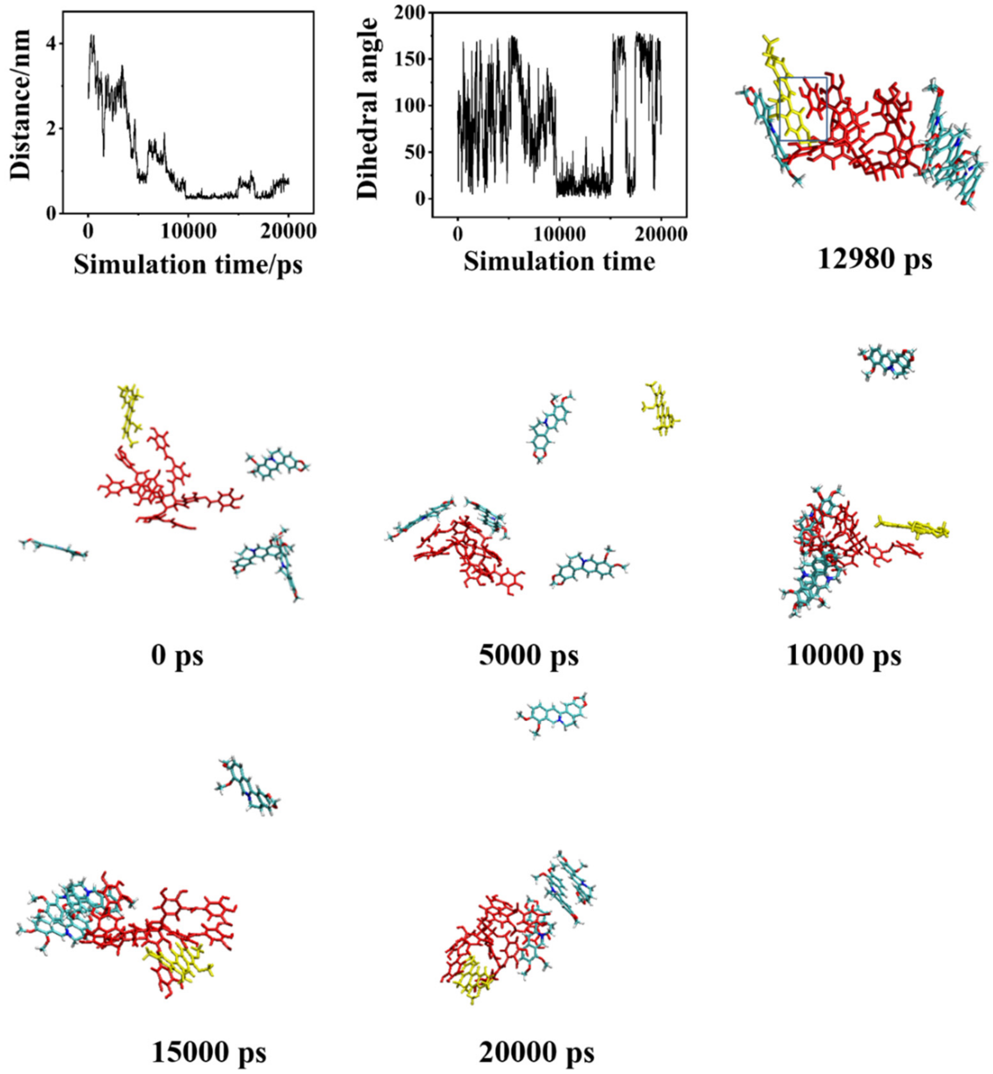

Figure S2. π-π conjugation of molecular simulation system of BBR and TA.

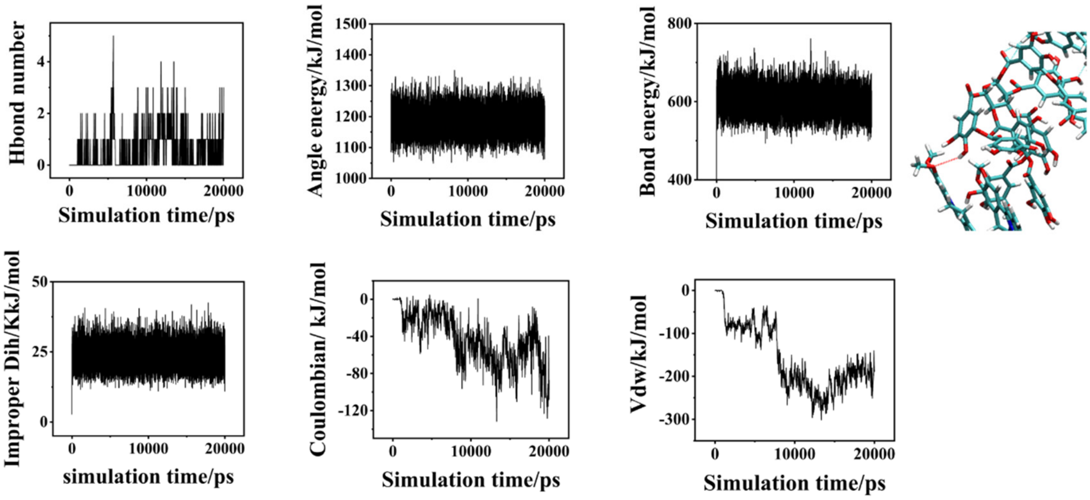

Figure S3. Hydrogen bonding of molecular simulation system of BBR and TA.
